# On the Use of Hydrobromic Ether as an Anæsthetic for Surgical Operations

**Published:** 1880-02

**Authors:** Lawrence Turnbull

**Affiliations:** Philadelphia; Aural Surgeon to Jefferson Medical College Hospital; Physician to the Department of Diseases of the Eye and Ear, Howard Hospital, Philadelphia; Fellow of the American Association for the Advancement of Science, and of the American Medical Association, etc.


ARTICLE III.

### ON THE USE OF HYDROBROMIC ETHER AS AN ANÆSTHETIC FOR SURGICAL OPERATIONS.

ABSTRACT OF A LECTURE DELIVERED FEBRUARY 6, 1880, AT THE PENNSYLVANIA COLLEGE OF DENTAL SURGERY,

BY LAURENCE TURNBULL, M. D., PH. G., PHILADELPHIA.

(Aural Surgeon to Jefferson Medical College Hospital; Physician to the Department of Diseases of the Eye and Ear, Howard Hospital, Philadelphia; Fellow of the American Association for the Advancement of Science, and of the American Medical Association, etc.)

REPORTED BY LEWIS S. AYER, D. D. S.

After discussing the subject of pain, its different meanings from the derivations of the word, and also as noted from its action or effect on the brain from the body, and vice versa, the lecturer spoke of the different articles which had been and were at the present time used for obtunding the sensibility of the nervous system.

Dr. Turnbull said, that in the summer of 1877 he had introduced the use of Hydrobromic Ether to the profession. He first experimented upon himself as to its advantages as an anæsthetic, and since that time he had employed a large quantity of it in numerous operations—in all, about one hundred cases. He and Dr. Levis had also brought it before the British Medical Association at Cork, and recommended its use as a stimulant in certain hysterical and nervous forms of deafness and noises in the ears, by introducing it as vapor into the middle ear, and also by administering it internally. The doctor also stated that he had read a paper before the Pharmaceutical Section at the International Congress at Amsterdam, and there proved the following facts in reference to it:

*First*—That it was safe as an anæsthetic, being rapid in its action, and also in its being eliminated from the body.

*Second*—It was much more agreeable in its odor and taste than ordinary ether, and, therefore, can be used in a private office or lady's chamber.

*Third*—The cost now is about thirty cents per ounce, and can be had pure of John Wyeth & Bro., and almost any ordinary case can be brought under its anæsthetic influence with half an ounce.

*Fourth*—Vomiting is very rare, unless the stomach has been recently filled to repletion.

*Fifth*—The vapor is not inflammable. Recently, in the Pennsylvania Hospital, Dr. R. J. Levis informed him he had employed the hydrobromic ether with perfect success in numerous protracted operations; also, he has had a letter from Chicago that Dr. Byford and one other surgeon of that city have used it with success in painful operations.

While conducting his investigations concerning the new anæsthetic, hydrobromic ether, or bromide of ethyl (bromic ether) which he introduced and recommended to the profession, he learned, through the courtesy of a brother professional who had been searching through some old medical literature, that, in an article upon "Anæsthesia and Anæsthetic Substances Generally," the late Dr. Thomas Nunnely, of Leeds, England,* reported, as far back as 1849, that he had employed the bromide of ethyl in experimenting upon animals, and in 1865, at the meeting of the British Medical Association,† Dr. Nunnely stated

* Transactions of Provincial Medical Association, Vol. 16, 1849, page 206.

† *British Medical Journal*, August 19, 1865, page 192.

that he had employed either hydrobromic ether or Dutch oil (chloride of olefiant gas) in all the principal operations at the Leeds Eye and Ear Infirmary.

Notwithstanding the publishing of these facts, promulgated at the meeting of such a society as we have mentioned, no further notice seems to have been taken of the subject. It was mentioned as an agreeable substitute for chloroform and ether, but not fully endorsed nor recommended alone, and all interest awakened by the anæsthetic, if there ever was any, died out, because from that time no mention of the facts under consideration was ever made until revived by the French in 1876 and by the writer. He was, however, not only the first one to use it in this country upon man, but also the first one in this country *to recommend, from his own experience*, its adoption for general use in surgery, *as both safe and most convenient;* and at the same time to point out its advantages over both chloroform and ether, or any other anæsthetic.

His first experiments, after having employed the lower animals, were made upon himself and subsequently upon surgical cases before he took the liberty of recommending hydrobromic ether* to the profession at large.

Thus coming to the subject in hand, three experiments were performed, viz: First, placing a pigeon under a glass frame of a little over a square foot in size, in which was a sponge saturated with the bromide of ethyl. It was seen that for some minutes the bird was not affected, but at about the *third* minute it seemed to lose the power of locomotion, and gradually settle down into a state of quietude for a few seconds, when it appeared to be somewhat nauseated, ejecting a few grains of corn, and then again settling to a quiet sleep. Thus it remained, the air being admitted to it freely for about two-and-a-half to three minutes, then suddenly flew around the room, entirely recovered.

While the bird was in the glass, a dog and rabbit were also placed under the influence, taking about two minutes for the rabbit, but as the dog succeeded in obtaining a small amount of air, it took a somewhat longer time. While under the influence there was slight tetanic movement of the extremities, and soon after removing the ether slightly stertorous breathing, from which, in the space of three to four minutes, the animal had entirely recovered consciousness, although it did not seem to be inclined to move.

*"Advantages and Accidents of Artificial Anæsthesia," Philadelphia, 1878.

The rabbit was apparently dead, but it is our opinion, had it been taken into fresh air, antidotal remedies and artificial respiration applied, it might have recovered, but these not being tried, it succumbed.

Upon making a post-mortem, the right side of the heart was found engorged with dark venous blood, the left side very much contracted and empty. The lungs were beautifully shown by inflation, and but very slightly congested at their base, *not enough* to have had *any* effect in causing death. The kidneys were also very much congested with *venous* blood. The brain was entirely free from blood, not even the puncta vasculosa being seen, but at the torcular Herophili was found a large drop of the same dark blood. The object of these experiments was to show, as a rule, the safety of this form of anæsthetic in all vivisections, and that it is not an irritant to the lungs.

